# Long-Term Effectiveness of Internet-Based Versus Traditional Cognitive Behavioral Therapy Across Psychiatric Disorders: A Systematic Review and Meta-Analysis

**DOI:** 10.1192/j.eurpsy.2026.10919

**Published:** 2026-07-31

**Authors:** R. Bakanaitė, I. Bakanienė

**Affiliations:** 1 Wellbeing Division, Vilnius University, Vilnius; 2 Lithuanian University of Health Sciences, Kaunas, Lithuania

## Abstract

**Introduction:**

Internet-delivered cognitive behavioral therapy (iCBT) has become a promising alternative to face-to-face CBT, yet its long-term effectiveness across psychiatric disorders remains unclear. While numerous studies have examined short-term outcomes, it is essential to understand how iCBT compares with traditional CBT over extended periods and which conditions benefit most.

**Objectives:**

This review aims to analyze RCTs that compare iCBT and face-to-face CBT within the same clinical trials, reducing potential effect size inflation and providing clearer insights into iCBT’s long-term effectiveness (12 months or more).

**RESEARCH QUESTIONS**

1. What are the long-term effects of iCBT compared with traditional CBT in reducing symptom severity in individuals with anxiety, depression, and eating disorders?
2. How do dropout rates differ between iCBT and face-to-face CBT across various psychiatric disorders, and what factors are most predictive?
3. Which psychiatric disorder among anxiety, depression, and eating disorders shows the most significant symptom improvement when treated with iCBT?

**Methods:**

A systematic search of PubMed, Embase, MEDLINE via EBSCO, PsycINFO via APA, Scopus, and Cochrane Library (CENTRAL) was conducted up to May 21, 2025. Randomized controlled trials (RCTs) comparing the long-term effects of iCBT and face-to-face CBT for depression, anxiety disorders, and eating disorders were included. The random-effects model was used to calculate the standardized mean difference (Hedges’ g), and a chi-square test assessed adherence differences.

**Results:**

Eleven RCTs (1,272 participants) met the inclusion criteria. Pooled analysis showed no significant difference in long-term effectiveness between iCBT and face-to-face CBT (Hedges’ g = −0.07, 95% CI: −0.36 to 0.21). Depressive disorders responded best to iCBT, while anxiety disorders showed mixed results, and eating disorders had the least favorable outcomes. Adherence was higher in face-to-face CBT (86.68%) than in iCBT (70.06%), with therapist-guided iCBT improving completion rates (79.09%) compared with self-guided formats (48.17%).

**Image 1:**
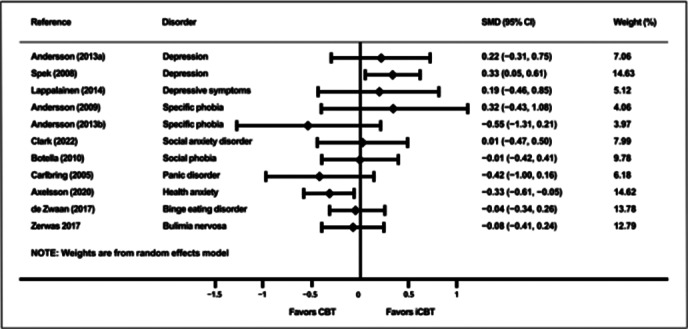
Image 1: Long description.

**Conclusions:**

iCBT is a promising alternative to traditional face-to-face CBT, demonstrating treatment effectiveness even in the long term. However, dropout rates are higher in iCBT, particularly in unguided formats, suggesting that therapist support may be crucial for ensuring participant adherence. Future research should optimize iCBT delivery, tailor it to specific disorders, and develop strategies to improve engagement.

**Disclosure of Interest:**

None Declared

## Long descriptions

### Image 1: Long description

A forest plot table with eleven rows of data. The x-axis at the bottom ranges from -1.5 to 1, with 0 as the line of null effect. Values to the left of 0 are labeled Favors C B T and values to the right are labeled Favors i C B T.

Data rows from top to bottom:

1. Andersson 2013a, Depression: S M D 0.22 (-0.31, 0.75), Weight 7.06 percent.

2. Spek 2008, Depression: S M D 0.33 (0.05, 0.61), Weight 14.63 percent.

3. Lappalainen 2014, Depressive symptoms: S M D 0.19 (-0.46, 0.85), Weight 5.12 percent.

4. Andersson 2009, Specific phobia: S M D 0.32 (-0.43, 1.08), Weight 4.06 percent.

5. Andersson 2013b, Specific phobia: S M D -0.55 (-1.31, 0.21), Weight 3.97 percent.

6. Clark 2022, Social anxiety disorder: S M D 0.01 (-0.47, 0.50), Weight 7.99 percent.

7. Botella 2010, Social phobia: S M D -0.01 (-0.42, 0.41), Weight 9.78 percent.

8. Carlbring 2005, Panic disorder: S M D -0.42 (-1.00, 0.16), Weight 6.18 percent.

9. Axelsson 2020, Health anxiety: S M D -0.33 (-0.61, -0.05), Weight 14.62 percent.

10. de Zwaan 2017, Binge eating disorder: S M D -0.04 (-0.34, 0.26), Weight 13.78 percent.

11. Zerwas 2017, Bulimia nervosa: S M D -0.08 (-0.41, 0.24), Weight 12.79 percent.

A note at the bottom states: Weights are from random effects model.

Navigate back to Image 1:.

